# Home or hospital as the place of end-of-life care and death: A survey among Chinese residents of Macao

**DOI:** 10.3389/fpubh.2023.1043144

**Published:** 2023-01-27

**Authors:** Kuai In Tam, Sok Leng Che, Mingxia Zhu, Sok Man Leong

**Affiliations:** Kiang Wu Nursing College of Macau, Complexo de Cuidados de Saúde das Ilhas—Edifício do Instituto de Enfermagem Kiang Wu de Macau, Avenida do Hospital das Ilhas No. 447, Coloane, RAEM, Macau, Macau SAR, China

**Keywords:** palliative care, preferred place of death, preferred place of end-of-life care, end-of-life decision making, palliative home care

## Abstract

**Objectives:**

The study was the first to explore Chinese residents' preferred place of care at the end of life and preferred place of death in Macao.

**Methods:**

A cross-sectional questionnaire survey was conducted online and face-to-face. The questionnaire was designed in Chinese, and both online and face-to-face surveys were conducted in Chinese. The study was conducted in Macao. Macao residents aged 18 years and older were recruited.

**Results:**

A total of 737 responses were valid, 65% were female, aged between 19 and 101 years; 43.4% of respondents preferred to be cared for at home in the last 6 months; however, less than one-fifth preferred to die at home. One-third of respondents chose to die in the hospice, and over a quarter of them preferred to die in hospitals. Compared with people aged between 18 and 39 years, people aged between 40 and 64 years did not want to be cared for at home in the last 6 months, and they did not want to die at home either.

**Conclusion:**

The results of the study suggested that there is a need for palliative home care in Macao, and the government should consider developing such a service and review current laws and regulations in supporting the service. Education is equally important for healthcare professionals, enabling them to support palliative care development in the community.

## 1. Introduction

Since the publication of the Quality of Death Index in 2015 (1), there has been an increasing focus on improving the level of comfort and the degree of autonomy people could have at the end of their lives. In addition to the accessibility of palliative care, the preferred place of death is an important component when considering the quality of death. As emphasized in the Quality of Death Index, the coherence between the preferred place of death and the actual place of death is a reflection of the quality of end-of-life care (1, 2). However, end-of-life care preference is often incongruent with the actual care received; as evidence suggests, people would prefer to die at home if a choice was presented (3–6), and yet, the results of many studies have shown that only a small number of patients died in their preferred place (7–9).

Place of death is undeniably an important quality marker for palliative care, and while the overall development of palliative care has garnered much emphasis, discussions relating to patients' preference for care, particularly their preferred place of death, have not received much attention. Concerning research on the place of death among the Chinese population, the majority of the studies were limited to the population of certain geographic locations, that is, Taiwan and Singapore (7, 10–12). This is not surprising as palliative care in both Taiwan and Singapore has been well-integrated into the mainstream healthcare system (1), and end-of-life decisions such as advance directives and advance care plans are legislatively recognized and protected in both places. Other studies addressing the issue of the place of death in mainland China focused on specific populations, such as patients with cancer who were terminally ill (13) and retrospective studies on individuals with cardiovascular diseases (14). There are limited studies on the general population concerning the preference for place of death among Chinese people, particularly in areas that are already under the impact of a rapidly aging population. Macao is one such place that has become an aging society since 1981, with over 12.1% of the population aged 65 years and older in 2021 (15). The population of Macao is predicted to age continuously, and by the year 2036, the 65 years and older will account for one-fifth of the overall population (16), qualifying Macao into an aged society in just over a decade. In addition to the aging population, Macao's life expectancy was 84.1 years in 2020 (17), and according to a previous study, over 80% of older adults in Macao had one or more chronic illnesses (18), implying the need for healthcare is considerably high for older people at the end of their lives. However, the current provision of palliative care lacks the infrastructure to cater to the changing needs of the changing demographic landscape of Macao. Macao's first hospice was established in the year 2000, offering both palliative and end-of-life care for people diagnosed with terminal cancer. Since the hospice only offers its services to people dying of cancer, for an extended period of time, people dying of other life-threatening illnesses neither receive any palliative care nor end-of-life care in Macao. The hospice remained the only inpatient service until 2019 when the government decided to establish another inpatient palliative care ward and open its services for people dying of illnesses other than cancer. Nevertheless, the provision of palliative care in Macao is currently limited to inpatient care, which certainly lacks the capacity to meet the need of the aging population as well as the increasingly complex health conditions among the population.

In terms of policy, the Macao Government has initiated an action plan with a specific focus to expand end-of-life care services in the community for older adults (44). However, described in the 10-year plan was general service improvement in the community and professional education with no specific goals (44). Furthermore, since the introduction of the 10-year action plan in 2016, there was no evaluation of the plan to check whether any real impact was made in improving and expanding end-of-life care services in the community.

The policy to expand community end-of-life care services and the current inpatient-only palliative care provision pose a mismatch between policy and practice, and both policy and practice are developed without taking into consideration of the real demand of the people in Macao.

Autonomy is a core element of palliative care, wherein people should be supported to make decisions relating to the care and treatment they wish to receive, including their preferred place of care and death. Pertaining to the government's goal of expanding end-of-life care in the community, people's autonomy to make decisions about their care and where they wish to be cared for at the end of life is crucial. Therefore, the current study aims to address this knowledge gap and focuses on people's preference for the place of end-of-life care and death.

Some researchers have contested that the place of death is not an inclusive metric for the quality of end-of-life (19); however, their argument is primarily based on high-income countries, wherein palliative and end-of-life care are already well-integrated into the mainstream healthcare system. However, Hoare et al.'s study did not take into consideration of places that do not have an integrative system of palliative care, and the concept of cure-oriented care is prevalent. While Macao has developed palliative inpatient services since the year 2000, palliative care service still remains limited, and there is currently neither community nor home palliative care available. The lack of choices in care certainly does not reflect people's lack of wishes for an alternative way to be cared for at the end of their lives. Hence, the current study sees it necessary to examine people's preference for the place of death, in order to demonstrate the need for further development in palliative and end-of-life care. The current study, therefore, aims to describe the preference for the place of death among the general Chinese population and to identify the potential factors associated with the preference for the place of death in Macao. The findings of this study may contribute to actualizing the improvement of end-of-life care services in Macao.

## 2. Methods

This study was a cross-sectional survey conducted between July and September 2020. The study employed convenience sampling according to the population statistics at the end of 2019 in Macao (20).

### 2.1. Participants

The inclusion criteria for participants were as follows: (i) individuals who were Macao residents aged 18 years or older, and (ii) individuals who could understand informed consent materials and questionnaire content of the study. The exclusion criteria were as follows: (i) individuals who have a severe hearing impairment and individuals who are unable to communicate, and (ii) individuals who may feel their levels of distress may hinder the capacity for informed consent and/or consider participation overwhelming. The survey was open to anyone who met the inclusion criteria to answer. The research team understood that people of different age groups would tend to have different preferences regarding the place of end-of-life care and dying; hence, the research team recruited Macao Chinese residents of different age groups for the study to ensure equal representation from all age groups. The current study therefore aimed to recruit participants in accordance with the Macao population distribution in the three age categories (18–39 years, 40–64 years, and 65 years and older) at the end of 2019. The means of obtaining responses were as follows: (1) The online questionnaire was sent to a couple of the social organizations to distribute to its members across different age groups. (2) The research team distributed the online questionnaires to friends and friends of friends; the questionnaire was also distributed by way of social media platforms. In the process of data collection, the team constantly monitored the number of questionnaires received in each age group, and the team continued to disseminate questionnaires to groups that needed more participants. The sample size for this survey study was calculated according to Charan and Biswas (21) and Pourhoseingholi et al.'s (22) sample size calculation for the survey study, and the minimal sample size for this study was 384, with a level of significance of 0.05, the absolute error of 5%, and at type 1 error of 5%.

### 2.2. Recruitment

In terms of recruitment, the study advertised and recruited respondents from tertiary education institutions, social media platforms, and three of the largest social organizations for adults with different backgrounds across different age groups; a self-administered electronic version of the questionnaire was completed through the online platform Survey Monkey (https://www.surveymonkey.com/). Participants were requested to answer all questions to move forward on the questionnaire platform. The participants were provided with a “back button” to amend their responses. For respondents aged 60 years or older, recruitment was also conducted through day centers for older adults, and the questionnaire was completed face-to-face by trained interviewers. Respondents of face-to-face interviews would receive a non-monetary incentive (eco-friendly shopping bags) for their voluntary participation.

### 2.3. Measurement

The current study is the first to explore Chinese residents' preferred place of care at the end of life and preferred place of death in Macao. Since there was no previous study of this kind, the research team developed our own structured questionnaire by referencing other existing studies (23–25). In developing the questionnaire, the research team organized an expert panel with three experts in palliative care from Beijing, Macao, and Hong Kong to assess the content validity of the questionnaire. This study used the content validity index (CVI) as the criterion for item revision or elimination (26). The experts scored 1 to 4 (completely irrelevant to completely relevant) according to the relevance of items to the study and gave recommendations for item revision. The items, which scored 1 and 2 were then converted to 0 and scored 3 and 4 to 1. Items with an item content validity index (I-CVI) lower than 0.8 suggested the need to be revised or eliminated. The research team also modified the content of the items according to experts' opinions. The score of I-CVI for this study questionnaire was 0.96, indicating that the items were relevant to the domain of inquiry. The survey was then pretested on 55 Macao residents recruited as per the inclusion and exclusion criteria mentioned earlier to check the readability of the revised questionnaire. The questionnaire included four sections: (1) Sociodemographic data including age, gender, education level, occupation, employment status, income, marital status, any children, and experiences of caring for relatives or friends with a terminal illness, implying whether participants had ever been involved in physically, emotionally, or financially cared for relatives or friends with a terminal illness, participants were offered to choose between “yes” or “no”, religious belief, regarding the options for religious belief, Christianity encompasses Protestantism and Catholicism, due to the relatively small sample size of this study, the research team had therefore decided to combine both religions into a single option as Christianity. In addition to the options provided in the questionnaire, the option “others” is also provided for respondents to express in text their religious beliefs; (2) Preferences for end-of-life treatment options and attitudes toward end-of-life care: the first question asked, “If the doctor has diagnosed you with an incurable illness, and you are estimated to have < 6 months to live, which of the following options would you choose?” Respondents were presented with four choices: (i) “I will accept all life-prolonging treatments, despite discomfort or sufferings that may occur during the treatment process”, (ii) “I will accept treatments that can ease pain and sufferings, or alleviate discomfort caused by symptoms, despite my life may not be extended”, (iii) “I don't know/ I don't have a decision”, and (iv) “Don't want to answer”. The second question asked, “If the doctor has diagnosed you with an incurable illness, and you are estimated to have < 6 months to live, would you agree that all life-sustaining treatments should not be stopped under any circumstances?”, respondents were then presented with five statements: (i) strongly agree, (ii) agree, (iii) neither agree nor disagree, (iv) disagree, and (v) strongly disagree. The Hospice Care Attitude Scale was used to assess respondents' attitudes toward end-of-life care. The scale was validated by the research team, and details of the development and validation of the scale were published in another study (27). The Hospice Care Attitude Scale has three dimensions, including psychological, biological, and social dimensions. The psychological dimension has five items, the biological dimension has four items, and the social dimension has two items, totaling 11 items. The Hospice Care Attitude Scale measures on a five-point scale from “very unimportant” to “very important”. The total score of the scale ranges from 11 to 55, the higher the score, the more positive attitude respondent has toward end-of-life care; (3). Preferred place of end-of-life care: “If the doctor has diagnosed you with an incurable illness, and you are now in the last stage of your life, where would you like to be cared for?” Respondents were presented with six choices: (i) Home, (ii) Nursing home/residential home, (iii) Hospital, (iv) Hospice/palliative care ward, (v) Others, and (vi) Do not want to answer, and respondents were asked to answer the question for three different time frames: (A) In the last 6 months of your life, (B) In the last few weeks of your life, and (C) In the last few days of your life; (4) Preferred place of death: “Where would you like to die?” Respondents were presented with six choices: (i) Home, (ii) Nursing home/residential home, (iii) Hospital,(iv) Hospice/palliative care ward, (v) Others, and (vi) Do not want to answer. For respondents who choose “home” as their preferred place of death, they were then presented with another question: “Do you have any concerns if you were to die at home?” with “yes” or “no” answering options. If respondents answered “yes”, and for all other respondents who had answered anywhere apart from “home” in the preferred place of death question, they were then led to the final question of the section: “What is your concern if you were to die at home?” Respondents were asked to choose their top three concerns from the eight choices provided: (A) lack of support from medical and nursing staff, (B) lack of care support, (C) concern property prices of their home would be affected, (D) complicated legal issue and procedure involved after death at home, (E) do not want to be a burden to family, (F) concern about neighbor's fear and unease, (G) compulsory autopsy of their bodies after death at home, and (H) others. There were a total of 32 items in this survey. The pilot test was completed to check the suitability of the questions and the process of answering the electronic questionnaire. The questionnaire was developed in Chinese, and the research process was carried out in Chinese. Since the questionnaire and all information regarding the research offered to participants were in Chinese, there was no translation issue during our research process. The questionnaire was only translated for publication purposes. The main author was proficient in both professional and academic English, and all translated materials were cross-checked.

### 2.4. Ethics

Ethics approval was obtained from the Kiang Wu Nursing College of Macau, and the reference number was 2019OCT01. The entire study was overseen by the research management and development department of the college. Informed consent was obtained from all participants before the commencement of the questionnaire. Detailed information about the study and the decision regarding consent to participate was provided on the first page of the online questionnaire. If participants agreed to participate in the study, they would choose the “understand and agree to partake” option, which would lead them to the online questionnaire page; for participants who did not wish to participate, they would choose “No, I do not wish to partake” option, which would lead them to a closing page, with a thank you message. All participants were required to give their informed consent before proceeding to the online questionnaire. During face-to-face interaction, the researchers would still use the online questionnaire and all participants would still be required to read through the information page about the study and gave their informed consent if they wished to participate. All collected data were stored in a digital file with a code accessible only to members of the research team and were recorded and stored on a computer. The computer and all files stored were password-protected.

### 2.5. Statistical analysis

Microsoft Office Excel 2013 was used for data input and coding. All analyses were conducted using SPSS 22.0 software. Descriptive statistics were performed to summarize the responses of the participants. Multiple logistic regression analysis was conducted by using the willingness to die at home as a dependent variable, and other associated items examined in *t*-tests as independent variables to identify predictors. The threshold for statistical significance in this study was set to *p* < 0.05, and independence of error was confirmed using a residual plot. Only respondents who completed the full questionnaire were considered valid for logistic regression.

## 3. Results

### 3.1. Demographic characteristics

The study received a total of 1,001 responses, and 737 were valid responses; within the valid responses, 635 responses were collected from a self-administered online questionnaire, and 102 were collected from face-to-face interviews; 65% of the respondents were female, and the age distribution reflects the population of Macao in 2019; 56.9% of the respondents had bachelor or higher level of education; 66.5% of respondents were married or cohabited; 74.1% of respondents were employed or self-employed, and 32.6% of those were working as professionals; 61.5% of respondents cared for relatives or friends suffering from terminal illnesses (Table 1). In terms of the Hospice Attitude Scale, the total score of the scale in this study was 47.13 ± 5.08, the corresponding score for the psychological dimension (five items) was 21.23 ± 3.02, the biological dimension (four items) was 17.88 ± 2.10, and the social dimension (two items) was 8.03 ± 1.57.

**Table 1:** Sociodemographic characteristics of participants (*N* = 737).

**Variables**	**n/M±SD**	**%**
**Gender**		
Male	258	35.0
Female	479	65.0
**Age**		
18–39	310	42.1
40–64	305	41.4
≥65	122	16.6
**Education**		
Primary school or below	96	13.0
Secondary school	216	29.3
Bachelor or above	419	56.9
Other	6	0.8
**Marital status**		
Unmarried	154	20.9
Married/cohabited	490	66.5
Separated/divorced	38	5.2
Widowed	55	7.5
**Children**		
Yes	519	70.4
No	218	29.6
**Religious belief**		
None	428	58.1
Christianity	121	16.4
Buddhism/ Chinese folk beliefs	188	25.5
**Employment status**		
Employed/ self-employed	546	74.1
Student	14	1.9
Unemployed	18	2.4
Housewife/househusband	30	4.1
Retired	129	17.5
**Occupation (*****n*** = **546)**		
Professional	178	32.6
Medical (assistant) professional	86	15.8
Technician	108	19.8
Attendant	133	24.4
Disciplined services	20	3.7
Other	21	3.8
**Average monthly income in the past year (MOP)**		
< 9,999	149	20.2
10,000–19,999	141	19.1
20,000–29,999	177	24.0
≥30,000	224	30.4
No answer	46	6.2
**Cared for relatives or friends suffering from terminal illness**		
Yes	453	61.5
No	284	38.5
**Self-rated health**		
Not good	432	58.6
Good	305	41.4
**Score of hospice care attitude scale**		
Total (11 items)	47.13 ± 5.08	
Psychological dimension (5 items)	21.23 ± 3.02	
Biological dimension (4 items)	17.88 ± 2.10	
Social dimension (2 items)	8.03 ± 1.57	
**Method of data collection**		
Face-to-face	102	13.8
Self-administered	635	86.2

### 3.2. Preferred place of end-of-life care and death

When participants were asked where would they like to be cared for in the last 6 months of their lives if the circumstances allowed them to choose a home, nursing/residential home, hospital, or hospice/palliative care ward, almost half of respondents (43.4%) decided that home would be the most preferred place for end-of-life care, and only around 12% of participants would like to be cared for in the hospital if they knew they had 6 months to live (Table 2). Despite the preference for home gradually dropped as the number of remaining days of their lives decreased, there were still almost 20% of respondents who would like to die at home (Table 2). Conversely, though the hospital was not initially preferred for end-of-life care, over 20% of people would choose to spend their last days and die there. The preference of place of care in the last 6 months of life and death categorized into age and gender are presented in Tables 3, 4. The hospice/palliative care ward was consistently preferred as a place for end-of-life care and death, and it was the most preferred place of death for respondents (33.1%). Nursing home/residential home was consistently the least preferred place to be cared for or to die in (see Table 2), and this preference could be explained by Chinese people's unwillingness to live in nursing homes or residential homes due to cultural reasons, which will be further explored in the discussion section.

**Table 2:** Respondents' preferred place of end-of-life care and death (*N* = 737).

	**Last 6 months of life**		**Last few weeks of life**		**Last few days of life**		**Death**	
	***n***	**%**	***n***	**%**	***n***	**%**	***n***	**%**
Home	320	43.4	285	38.7	253	34.3	147	19.9
Nursing home/residential home	25	3.4	20	2.7	12	1.6	14	1.9
Hospital	89	12.1	120	16.3	150	20.4	214	29.0
Hospice/Palliative care ward	214	29.0	241	32.7	260	35.3	244	33.1
Others	30	4.1	20	2.7	11	1.5	23	3.1
Don't want to answer	59	8.0	51	6.9	51	6.9	95	12.9

If the doctor has diagnosed you with an incurable illness, and you are now in the last stage of your life, where would you like to be cared for?

**Table 3:** Respondents' preferred place of end-of-life care and death by age groups (*n* = 737).

	**Home**		**Nursing home/**		**Hospital**		**Hospice/Palliative**		**Others**		**Don't want**	
			**residential home**				**care ward**				**to answer**	
	***n***	**%**	***n***	**%**	***n***	**%**	***n***	**%**	***n***	**%**	***n***	**%**
**Last 6 months of life**												
18–39	148	47.7	7	2.3	27	8.7	90	29.0	7	2.3	31	10.0
40–64	110	36.1	6	2.0	33	10.8	112	36.7	20	6.6	24	7.9
>=65	62	50.8	12	9.8	29	23.8	12	9.8	3	2.5	4	3.3
**Death**												
18–39	73	23.5	3	1.0	74	23.9	105	33.9	12	3.9	43	13.9
40–64	40	13.1	6	2.0	89	29.2	126	41.3	8	2.6	36	11.8
>=65	34	27.9	5	4.1	51	41.8	13	10.7	3	2.5	16	13.1

**Table 4:** Older respondents' preferred place of end-of-life care and death by gender (*n* = 122).

	**Home**		**Nursing home/**		**Hospital**		**Hospice/Palliative**		**Others**		**Don't want**	
			**residential home**				**care ward**				**to answer**	
	***n***	**%**	***n***	**%**	***n***	**%**	***n***	**%**	***n***	**%**	***n***	**%**
**Last 6 months of life**												
Male	13	38.2	2	5.9	10	29.4	5	14.7	2	5.9	2	5.9
Female	49	55.7	10	11.4	19	21.6	7	8.0	1	1.1	2	2.3
**Last few weeks of life**												
Male	10	29.4	3	8.8	12	35.3	6	17.6	1	2.9	2	5.9
Female	36	40.9	7	8.0	29	33.0	7	8.0	2	2.3	7	8.0
**Last few days of life**												
Male	10	29.4	2	5.9	12	35.3	8	23.5	0	0.0	2	5.9
Female	31	35.2	4	4.5	38	43.2	9	10.2	0	0.0	6	6.8
**Death**												
Male	10	29.4	2	5.9	13	38.2	5	14.7	0	0.0	4	11.8
Female	24	27.3	3	3.4	38	43.2	8	9.1	3	3.4	12	13.6

### 3.3. Concerns regarding dying at home

As presented in Table 5, participants were asked their top three concerns regarding dying at home, and the most concerned issue being (1) don't want to be a burden to family (78.6%), (2) lack of assistance from the medical and nursing staff at home (41.2%), and (3) neighbor's fear and unease (33.2%). Respondents were also concerned about the lack of care assistance at home, and the potential legal issues involved after dying at home.

**Table 5:** Concerns regarding dying at home (*n* = 527).

**Item**	***n***	**%**
Don't want to be a burden to family	414	78.6
Lack of assistance from medical and nursing staff at home	217	41.2
Neighbor's fear and unease	175	33.2
Lack of care assistance at home	163	30.9
Complicated legal issue and procedure involved after death at home	149	28.3
Property prices of their home would be affected	93	17.6
Compulsory autopsy of their bodies	46	8.7
Others	18	3.4

Do you have any concerns if you were to die at home?

### 3.4. Predictors of home as the preferred place of death

The study examined the independent predictors of home as the preferred place of death. As shown in Table 6, compared with people in the age group of 18–39 years, people aged between 40 and 64 years were found to be 48% less likely to prefer a home as their place of death. Regarding the hospice care attitude, with every extra point attained, respondents were associated with a 4% increased likelihood to prefer to die at home. Caring for relatives/friends, self-rated good health, having children, and preference for palliative care was found to have no significant association with the preference for home as the place of death.

**Table 6:** Multiple logistic regression models of predictors of home as the preferred place of death (*n* = 686).

	***B***	**SE**	**Wald**	***P*-value**	**Exp (*B*)**	**95% CI for Exp (** ***B*** **)**	
						**Lower**	**Upper**
Constant	−1.99	1.22	2.64	0.10	0.14		
Female (ref.: male)	−0.13	0.23	0.29	0.59	0.88	0.56	1.39
**Age (ref.:18–39 years)**							
40–64 years	−0.66	0.26	6.66	0.01	0.52	0.31	0.85
≥65 years	−0.23	0.62	0.14	0.71	0.79	0.23	2.68
**Education (ref.: Primary school or below)**							
Secondary school	−0.86	0.56	2.42	0.12	0.42	0.14	1.25
Bachelor or above	−0.75	0.61	1.51	0.22	0.47	0.14	1.57
**Marital status (ref. unmarried)**							
Married/cohabited	0.27	0.37	0.52	0.47	1.31	0.63	2.70
Separated/divorced	0.35	0.58	0.36	0.55	1.42	0.45	4.46
Widowed	0.80	0.55	2.15	0.14	2.23	0.76	6.49
**Children (ref.:** **none)** **Religious belief (ref.: none)**	−0.16	0.33	0.24	0.63	0.85	0.44	1.63
Christianity	0.09	0.29	0.09	0.76	1.09	0.61	1.95
Buddhist/ Chinese folk beliefs	−0.04	0.24	0.03	0.86	0.96	0.59	1.54
**Occupation (ref.: Disciplined services/other)**							
Professional	−0.32	0.46	0.49	0.48	0.72	0.29	1.79
Medical (assistant) professional	−0.01	0.50	0.00	0.99	0.99	0.37	2.66
Technician	−0.64	0.51	1.55	0.21	0.53	0.19	1.44
Attendant	−0.11	0.49	0.05	0.83	0.90	0.34	2.35
Not employed	−0.85	0.56	2.27	0.13	0.43	0.14	1.29
**Average monthly income in the past year (ref.:**<**9,999)**							
10,000–19,999	−0.23	0.42	0.30	0.58	0.79	0.34	1.82
20,000–29,999	−0.35	0.42	0.71	0.40	0.70	0.31	1.59
≥30,000	−0.69	0.44	2.44	0.12	0.50	0.21	1.19
**Cared for relatives/** **friends (ref.: no)** **Good Self–rated health** **(ref.: not good)**	0.07	0.21	0.11	0.74	1.07	0.71	1.61
	−0.01	0.20	0.00	0.94	0.99	0.66	1.47
**Prefer palliative care (ref.:** **life prolongation)**	0.25	0.21	1.37	0.24	1.28	0.85	1.94
**Hospice care attitude**	0.04	0.02	4.27	0.04	1.04	1.00	1.09

## 4. Discussion

Our findings show almost half of our study respondents preferred to be cared for at home in the last 6 months of their lives, and over a third of the study respondents still would prefer to stay at home even in the last few weeks of their lives. However, as the number of remaining days of life decreased, the preference for home also gradually decreased while the preference for hospital increased. Consistent with existing studies (24, 28, 29), our study found that respondents tended to change their preference between the place of care and place of death, and some people would prefer to be cared for at home before they died. The wish to be cared for at home at the end of life indicates a demand for palliative home care services in the community; however, home care services in Macao are currently very limited. According to the Social and Welfare Bureau, home care service only entails a small range of nursing interventions, that is, maintenance of personal hygiene, wound care, nasogastric tube maintenance, and urinary catheter maintenance (30); regarding palliative home care, it is limited to consultation-based services offered by the hospice (31). Concern over the limitation of home care services was evident in our study findings; the second most important concern respondents had was the lack of professional medical and nursing care support at home if they were to die at home, and the lack of care assistant at home was also within the top five concerns among our study respondents. This finding is consistent with studies of the neighboring region where the lack of sufficient care at home was identified as a deterrent to home as the preferred place of death (24, 32). The concern of lack of home care is reflected in the changing preference, and as the findings of our study showed, the number of respondents who preferred to die at home dropped by more than half, and the number of respondents who preferred hospital as the place of death had more than doubled. According to a national study based in Singapore, receiving home palliative care was found to contribute to a higher possibility of home death (33). In Taiwan, it was also found that community-based palliative home care would facilitate patients' likelihood of dying at home by reducing emergency room visits (34). The significance of home palliative care in supporting home death was highlighted in the findings of the Cochrane review, which found access to home palliative care would more than double the likelihood of people dying at home (35), and when the home was the most preferred place of death, the availability of home palliative care was found to enhance the congruence between the preferred and actual place of death (36). As illuminated by our study findings, the current lack of home end-of-life care in Macao seemed to have inhibited people from choosing to receive care and die at home despite their wish to.

Another factor inhibiting home death was potentially related to the legal complication of dying at home. In Macao, for any cadavers found outside of hospital settings, mandatory criminal inquiry and post-mortem are necessary (37, 43), meaning that the majority of people in Macao would only be permitted a legally uncomplicated death in hospital settings while dying at home could have legal consequences. Despite the exemption from the post-mortem can be applied, the applicant (usually a family member of the deceased) still has to go through the lengthy and distressing process of criminal investigation, all witnesses and personnel involved are required to provide legal testimonies and potential court appearances, causing more trauma to the family after death. Nevertheless, the criminal inquiry is mandatory in all deaths that occur outside of the hospital, has inevitably posed legal restrictions on people's choice regarding their place of death and the legal complication of home death would concern people and would potentially deter people from choosing to die at home, as reflected in our study findings (see Table 5).

Considering the preference between the place of care and place of death can change over time (38), discovered in this study, is the consistent demand for palliative care from the last 6 months of life up to death; respondents consistently preferred to be cared for and to die in hospice or palliative care inpatient settings. In Macao, there is currently one inpatient hospice (35 beds) (31) offering palliative care only for people with terminal cancer, and a separate palliative care ward (20 beds) (39) offering care for people with other terminal conditions. Nevertheless, the existing palliative care service is limited, and most deaths in Macao continue to happen in acute hospitals (15), which further widens the gap between people's preferred place of death and their actual place of death.

The balance between people's preference to be cared for at home at the end of life and to die at home indicates a strong demand for not only just inpatient palliative care but also the infrastructure of outpatient and community palliative care. Highlighted in the Quality of Death Index in 2015, as countries are faced with rapidly aging populations and the healthcare system is under unprecedented pressure, the integration of palliative care in community care is pivotal to ensure people receive quality care at the end of their lives and a good death wherever they wish to (1, 34, 35). Considering the wider Chinese context, namely, mainland China, is also experiencing a rapidly aging population and taking into account the recent significance placed on introducing and developing palliative and end-of-life care in mainland China (40), the finding of this study illuminates the critical role of end-of-life care at home and the infrastructure of community palliative care.

In addition to our study, the number of respondents who preferred to be cared for or to die in the nursing home/residential home was significantly lower when compared with existing studies (4, 24, 41). The hesitation in choosing a nursing home could be related to the cultural belief and practice of filial piety, in that, looking after aging members of the family at home is traditionally recognized as a significant social norm in Chinese society (42), implying it is considered socially unacceptable to place senior members in nursing homes. Hence, the low preference for nursing home/residential home observed in our study is understandable given that the nursing home or residential home is not considered a desirable place to live as one ages. Furthermore, considering participants of our study were the general population of all ages, the option of nursing home/residential home would seem less appropriate for younger respondents. For instance, respondents who might be 20 years old at the time they answered the questionnaire might not consider the nursing home/residential home as an appropriate place for their death.

A unique finding from our study is that compared with people aged 18–39 years, 65 years and older, people aged between 40 and 64 years neither want to be cared for at home in the last 6 months nor want to die at home. Our finding is different from another study in Hong Kong in which people's preference to die at home had continued to decrease from the age of 40 years onward (24). Taking into consideration that people from the age group of 40–64 years usually bear a huge amount of social and family responsibilities, these responsibilities compounded with career pressure at this point of life might further reinforce their concern about not wanting to be a burden to their families, hence not wanting to be cared for at home or to die at home. The reality that Macao lacks palliative care, both at home and within the community, supports our study findings in that people were reluctant to die at home despite wishing to be cared for at home at the end of their lives.

In spite of a common concern that having death at home may affect property prices, similar to a previous study (24), our study respondents did not recognize this as their biggest concern; however, the fear of upsetting neighbors was among the top three concerns respondents had in dying at home. Hence, the findings of our study illuminate the need for public education in normalizing death and, at the same time, the need to build the infrastructure to support end-of-life care and dying at home. The findings of the study suggested that the Government of Macao should urgently refer to the experience of Taiwan and Hong Kong and enforce collaboration with healthcare professionals to comprehensively review and revise existing regulations, to enhance the feasibility of dying at home. Furthermore, resources should be invested in developing palliative care, particularly community palliative care and professional training in palliative care.

## 5. Limitations

Although the data were collected in 2020, the status of palliative care in Macao has not experienced any new development; hence, the authors were confident that the data would still be relevant to the questions asked in this research. While convenience sampling was adopted in this study, it was ensured that the age distribution of participants was comparable to the Macao population, and additional caution was applied in the analysis and interpretation of the data. The proportion of female participants in our study was higher than the female proportion of the Macao population, which may potentially restrict our sample representativeness. Our study primarily collected data from an online survey, which may have led to a relatively higher education tendency of our participants. Cross-sectional studies such as ours may not be able to capture all elements involved in the decision-making process related to the preference for end-of-life care and place of death. For the online questionnaire, the IP address was not checked to protect the confidentiality of participants, the authors, therefore, could not confirm whether one IP address might have been used two times. The current study focused on analyzing the impact of participants' hospice care attitude on end-of-life treatment decisions, and based on the limitation of the questionnaire and the consideration of answering time, the current study could not cover all influential factors. All participants of this study had provided their informed consent to participate in this study. Despite the authors having made all efforts to ensure all participants understood all questions included in the questionnaires, the authors recognized there could be a possibility that participants might have misunderstood the questions. Regarding the COVID-19 pandemic in Macao, the overall impact of the COVID-19 pandemic on the society of Macao was relatively temperate. Hence, the authors were contented that the impact of COVID-19 might have on participants' responses, and the interpretation of the study findings was minimal.

## 6. Conclusion and implications

Our study was the first to examine the general public's preference for the place of end-of-life care and death in Macao. Despite the fact that the majority of people would die in acute hospital settings currently, our study found that the demand to receive end-of-life care at home was high, and there was a small percentage of people who would prefer to die at home. Furthermore, it is clear from our study that there was a constant demand for palliative care. Considering one of the key indicators of good quality of death is the accessibility of care as, and when it is needed, our results implied that there is still a huge gap within the healthcare system of Macao in providing adequate care that could meet the needs and demands of people. Our study illuminated the urgent need for the government to expand the current palliative care services, particularly in the realm of home care, to support people's wish to be cared for at home at the end of their lives. Policy development and education for healthcare professionals are equally important to enable them to support palliative care development in the community. Future research on the construction of palliative home care infrastructure is needed to integrate palliative care in the community, ensuring people's end-of-life wishes are being respected and quality end-of-life care is being delivered.

## Data availability statement

The raw data supporting the conclusions of this article will be made available by the authors, without undue reservation.

## Ethics statement

The studies involving human participants were reviewed and approved by Kiang Wu Nursing College of Macau. The patients/participants provided their written informed consent to participate in this study.

## Author contributions

KIT, SML, SLC, and MXZ: study concept and design, analysis and interpretation of data, and critical revision of the manuscript for important intellectual content. KIT, SML, and SLC: acquisition of data. KIT and SLC: drafting of the manuscript. All authors' specific areas of contribution are listed above.
